# A Preliminary Study of the Effects of pH upon Fluorescence in Suspensions of *Prevotella intermedia*

**DOI:** 10.1371/journal.pone.0158835

**Published:** 2016-07-21

**Authors:** Christopher K. Hope, Karen Billingsley, Elbert de Josselin de Jong, Susan M. Higham

**Affiliations:** 1 Department of Health Services Research, University of Liverpool, Liverpool, United Kingdom; 2 School of Dentistry, University of Liverpool, Liverpool, United Kingdom; 3 Department of Medical Microbiology, Royal Liverpool and Broadgreen University Hospital, Liverpool, United Kingdom; 4 Inspektor Research Systems BV, Amsterdam, The Netherlands; The Forsyth Institute, UNITED STATES

## Abstract

The quantification of fluorescence in dental plaque is currently being developed as a diagnostic tool to help inform and improve oral health. The oral anaerobe *Prevotella intermedia* exhibits red fluorescence due to the accumulation of porphyrins. pH affects the fluorescence of abiotic preparations of porphyrins caused by changes in speciation between monomers, higher aggregates and dimers, but this phenomenon has not been demonstrated in bacteria. Fluorescence spectra were obtained from suspensions of *P*. *intermedia* that were adjusted to pHs commensurate with the range found within dental plaque. Two fluorescent motifs were identified; 410 nm excitation / 634 nm emission (peak A) and 398 nm excitation / 622 nm emission (peak B). A transition in the fluorescence spectra was observed from peak A to peak B with increasing pH which was also evident as culture age increased from 24 hours to 96 hours. In addition to these ‘blue-shifts’, the intensity of peak A increased with pH whilst decreasing with culture age from 24 to 96 hours. A bacterium’s relationship with the local physiochemical environment at the time of image capture may therefore affect the quantification of dental plaque fluorescence.

## Introduction

The oral anaerobe *Prevotella intermedia* exhibits red fluorescence due to the accumulation of porphyrins, specifically protoporphyrin IX (PPIX) [1]. Cultures of *P*. *intermedia* contain a mixture of different porphyrins which mostly comprises of Fe-protoporphyrin IX (hematin; not fluorescent) [2] together with fluorescent, iron-free protoporphyrin IX (83%) and coproporphyrin III (17%) [3]. A pioneering study of black-pigmented bacteria isolated from the oral cavity revealed that the fluorescent properties of *Prevotella intermedia* changed over time from ‘red-orange’ at 24–48 hours, ‘pink-orange’ after 7 days to ‘orange’ at 14 days in colonial growth; other oral anaerobes behaved similarly [4]. Although the cause for the observed changes in fluorescence as a function of time was not discussed, it was likely due the changing physicochemical environment within the bacterial colony, such as pH, the relative proportion of different porphyrins and overall porphyrin concentration.

Protoporphyrin IX conforms to the common absorption characteristics reported in other tetrapyrroles with an absorbance maximum (λ_abs_) at ~400 nm known as the Soret band (or B-band) which is complemented by smaller absorbance peaks at higher wavelengths (Q-bands) [5]. These absorption spectra are echoed in fluorescence spectrophotometry studies, which reported similar excitation maxima (λ_ex_) with a subsequent fluorescence emission maxima (λ_em_) ranging from 632.7 to 636 nm via the Q(0,0) transition [6,7,8,9]. The peak λ_em_ for protoporphyrin IX occurs together with a second, smaller emission peak at ~700 nm via the Q(0,1) transition known as the J-band which is also associated with the result of the formation of J-aggregates at intermediate pH when the porphyrin molecules self-aggregate [10,11]. The exact λ_abs_, λ_em_ and λ_ex_ values reported for a particular porphyrin study depended upon the physicochemical properties of that mixture; specifically the solvent, concentration of porphyrin [12] and pH [13,14]. The fluorescence properties of the porphyrin can change either as a subtle bathochromic (red) / hypsochromic (blue) shift in the position of the Soret and Q-bands [15] whilst more radical changes in spectra occur as a result of transformations in porphyrin conformation due to the formation of J- and H- aggregates [10,16,17] and shifts in the dimerization equilibrium [18,19]. Furthermore, the removal of iron from the haem molecule to leave behind fluorescent protoporphyrin IX [20] would result in an hyperchromic shift (increase in fluorescence intensity). Such changes in the fluorescence of salivary protoporphyrin IX at 405 nm excitation have recently been suggested to be a potential tool to aid in the diagnosis of oral cancer [21]. The fluorescence dynamics within bacterial cultures is further complicated by the presence of other non-fluorescent porphyrins (i.e. haem), which also absorb strongly at ~400 nm as well as water soluble porphyrins such as coproporphyrin and uroporphyrin [22]. The fluorescent properties of coproporphyrin found in *P*. *intermedia* are λ_ex_ 397 nm with λ_em_ 623 nm [6,23].

A bacterium’s relationship with the local physiochemical environment may therefore affect the quantification of dental plaque fluorescence using photographic methods, such as quantitative light-induced fluorescence (QLF) [24]. In order to study this possibility it was considered important to establish how local pH affected red fluorescence emissions *at the point of measurement* in an oral bacterium. This approach was intended to obviate any changes in fluorescence that may be due to different antecedent growth conditions and how this subsequently affects porphyrin metabolism or dynamic shifts within a multispecies community. Although the effects of pH upon the fluorescence of porphyrins has already been studied in an innovative series of experiments [25], this phenomenon has not yet been reported in a bacterial system.

A pilot fluorescence spectroscopy study showed that adding microlitre volumes of concentrated C_2_H_4_O_2_ (acetic acid) or NaOH (sodium hydroxide) to a cuvette containing a suspension of *P*. *intermedia* in phosphate-buffered saline affected the resulting fluorescence emissions (unpublished data). A more detailed study was planned to evaluate the effects of culture age and pH upon the fluorescence properties in cell suspensions of *P*. *intermedia* as a model of microbial fluorescence without the potentially confounding effects of phosphate buffering. The aim of this study was to evaluate if culture age and / or pH were important determinants in the quality of fluorescent light emissions in an oral bacterium and by extrapolation potentially inform the assessment of dental plaque biofilm by fluorescent imaging techniques such as QLF.

## Materials and Methods

### Sample Preparation

Stocks of *Prevotella intermedia* (American Type Culture Collection 25611) were maintained on anaerobic basal agar (Oxoid) supplemented with 5% defibrinated horse blood (TCS Biosciences, Botolph Claydon, UK) in an anaerobic chamber (80% N_2_, 10% CO_2_,10% H_2_) (Don Whitley, Shipley, UK) at 37°C. A swab of growth from a stock plate (1–3 days old) was transferred to a thickly-poured, fresh anaerobic basal agar plate and incubated for 24 hours, following which fresh bacterial growth was suspended by cotton swab into 5 ml of saline solution (Oxoid) and vortex mixed. The optical density of the cellular suspension was adjusted to 0.1 units at 650 nm with further saline, as measured by spectrophotometry (Model 2021, Cecil Instruments, Cambridge, UK), to a volume of no less than 30 ml. The standardised cell suspension was split into three aliquots of 10 ml; the first was unmodified, the second was supplemented with 10 μl of 0.05 M NaOH whilst the third was supplemented with 10 μl of 0.1 M NaOH. Such small volumes of concentrated alkali were employed to minimise dilution of the samples. The three aliquots were dubbed, ‘acidic’ (unmodified), ‘neutral’ (0.05 M NaOH) and ‘alkaline’ (0.1 M NaOH). A fraction of each aliquot was then taken to measure the pH (Jenway, Stone, UK). The swab sampling process was repeated on regions of undisturbed growth *from the same anaerobic basal agar plate* at 48, 72 and 96 hours following initial inoculation.

### Fluorescence Spectroscopy

3 ml of each sample was transferred to UV grade optical methacrylate cuvettes (Kartell, Noviglio, Italy) alongside a saline blank for analysis in a fluorescence spectrophotometer (Cary Eclipse, Agilent, Stockport, UK).) The scan parameters used to acquire excitation emission matrices (EEM) were as follows; excitation 390–450 nm, emission 480–750 nm, excitation slit 5 nm, emission slit 5 nm, scan rate 1200 nm/min, averaging time 0.1 s, emission data interval 2 nm. Additional scans were undertaken to focus on Q-band absorbance; excitation 380–600 nm, emission 633 nm (fixed), excitation slit 2.5 nm, emission slit 20 nm, scan rate 600 nm/min, averaging time 0.1 s, emission data interval 2 nm. The resulting 3 dimensional scan data (excitation wavelength vs. emission wavelength vs. fluorescence intensity) were exported to a spreadsheet program (Microsoft Excel) for analysis.

Specific excitation and emission spectra were then isolated from the EEM based upon the two subsequently identified peak wavelengths at acidic, neutral and alkaline pHs at 24, 48, 72 and 96 hour time points. These were termed peak A (410 nm excitation, 634 nm emission) and peak B (398 nm excitation, 622 nm emission). To limit the impact of the inherent signal-to-noise ratio at the chosen scanning parameters with respect to the numerical peak fluorescence values, an array of data 2 nm wide was prepared for peak A_410:634_ which incorporated 409, 410 and 411 nm excitation (3 values) and 634 and 636 nm emission (2 values; restricted by the scanning resolution) and likewise for peak B_398:622_ at 397–399 nm excitation and 622 and 624 nm emission. The average of these six fluorescence values was calculated at each time point and at each pH.

## Results

The pH of the ‘acidic’ suspensions of *P*. *intermedia* ranged from 5.32–5.74, ‘neutral’ from 6.78–7.27 and ‘alkaline’ from 7.84–8.63 (Table 1). There was a discrete distinction between the three different samples at any given time point and there was no overlap with other target pH values at other time points. The mean difference between acidic and neutral samples was 1.6 pH units and 1.3 pH units between neutral and alkaline samples. A parallel study [26] demonstrated that similar cell suspensions of *P*. *intermedia* at an optical density of 0.1 units at 650 nm contained 7.6 ± 0.2 log_10_ cfu ml^-1^.

**Table 1:** Measured pH of the *P*. *intermedia* suspensions

Sample	Composition	Time (h)	24	48	72	96	STDEV
**Acidic**	Bacteria + 10 ml Saline		5.74	5.32	5.36	5.54	0.19
**Neutral**	Bacteria + 10 ml Saline + 10 μl 0.05 M NaOH	**pH**	7.14	6.78	6.98	7.27	0.21
**Alkaline**	Bacteria + 10 ml Saline + 10 μl 0.1 M NaOH		8.63	8.00	7.84	8.48	0.38

**Fig 1:**
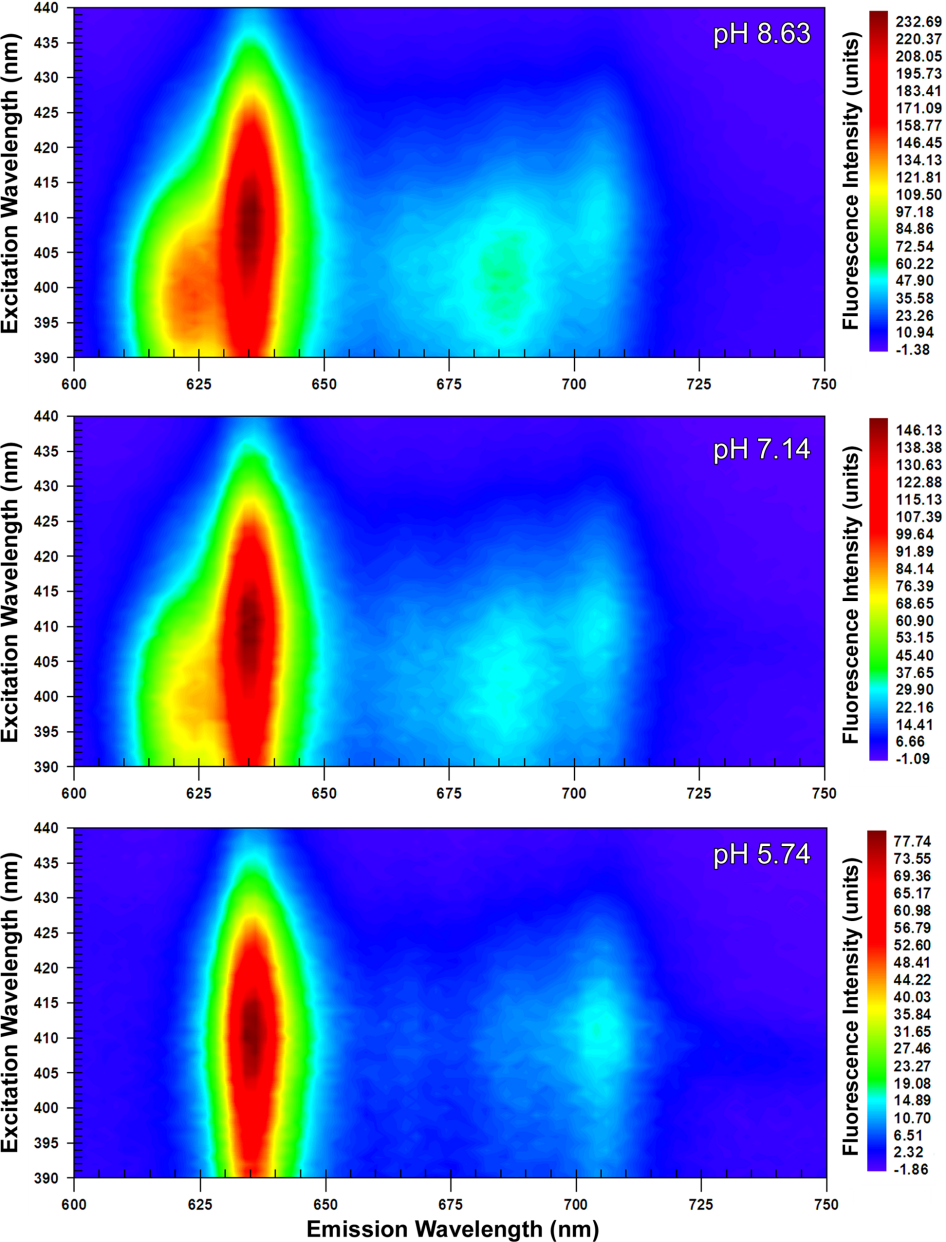
Excitation emission matrices of suspensions of *P*. *intermedia* after 24 hours growth. Adjusted to acidic (5.74), neutral (7.14) and alkaline (8.63) pHs. Peak A_410:634_ was predominant at acidic pH with a shoulder corresponding to peak B_398:622_ appearing at higher pHs.

**Fig 2:**
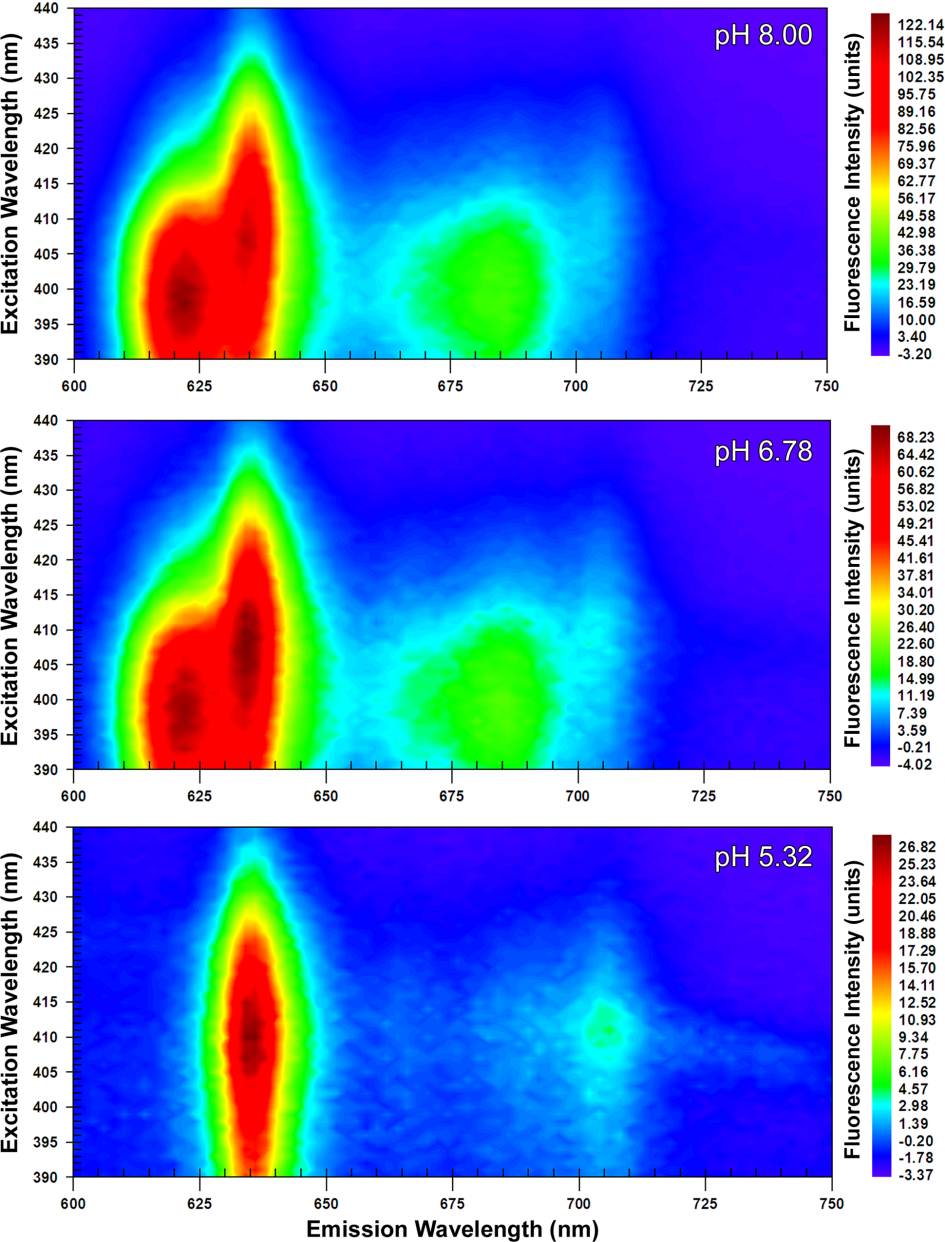
Excitation emission matrices of suspensions of *P*. *intermedia* after 48 hours growth. Adjusted to acidic (5.32), neutral (6.78) and alkaline (8.00) pHs. Peak A_410:634_ was predominant at acidic pH with shift towards peak B_398:622_ at higher pHs.

**Fig 3:**
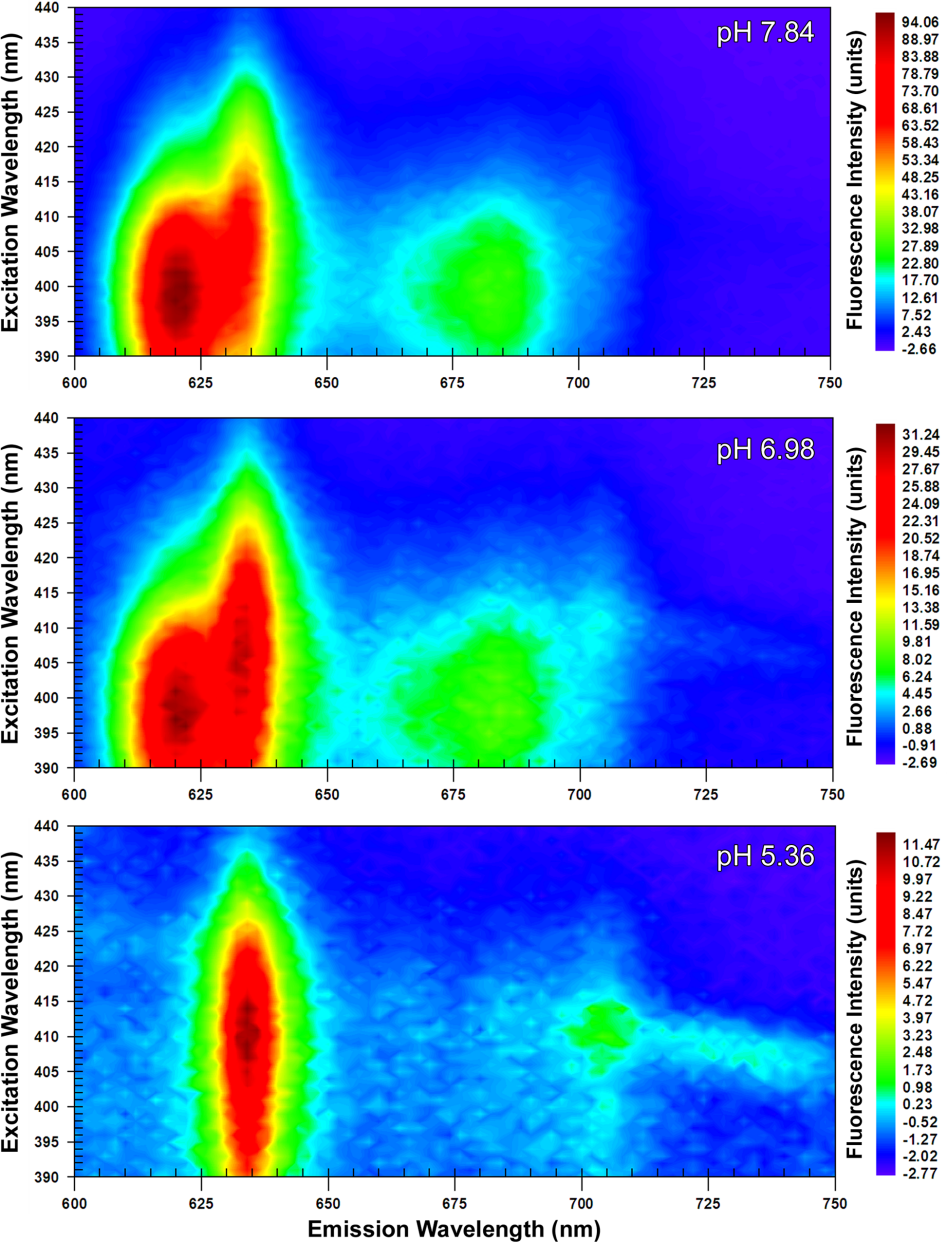
Excitation emission matrices of suspensions of *P*. *intermedia* after 72 hours growth. Adjusted to acidic (5.36), neutral (6.98) and alkaline (7.84) pHs. Peak A_410:634_ was apparent at acidic pH with peak B_398:622_ becoming dominant at higher pHs.

**Fig 4:**
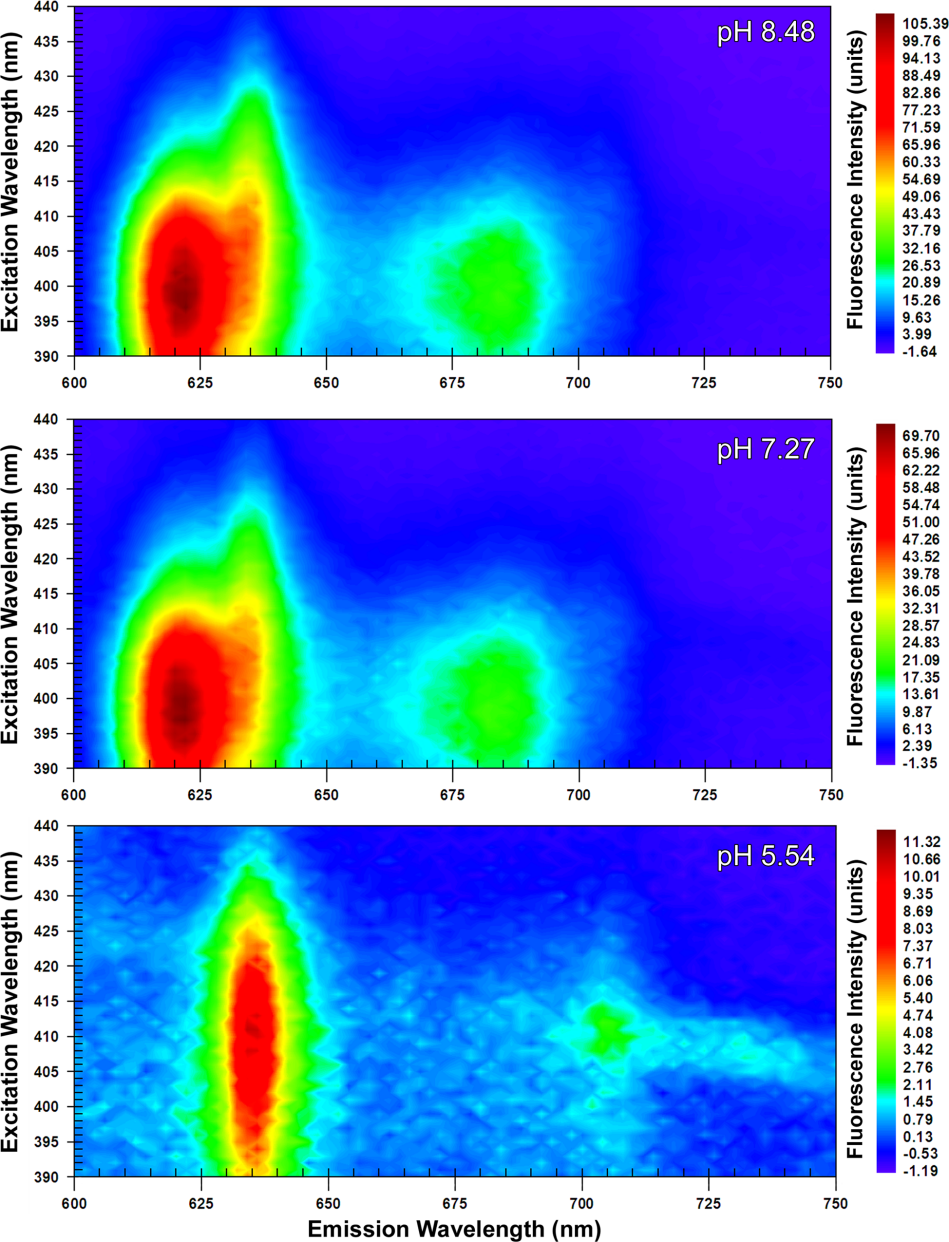
Excitation emission matrices of suspensions of *P*. *intermedia* after 96 hours growth. Adjusted to acidic (5.54), neutral (7.27) and alkaline (8.48) pHs. Peak A_410:634_ was apparent at acidic pH with peak B_398:622_ becoming dominant at higher pHs.

The EEMs captured at ‘acidic’, ‘neutral’ and ‘alkaline’ pHs revealed two dominant peaks at 410 nm λ_ex_; 634 nm λ_em_ (peak A_410:634_) and at 398 nm λ_ex_; 622 nm λ_em_ (peak B_398:622_). From 24 hour old colonial growth, the EEMs showed that peak A_410:634_ was dominant with a ‘shoulder’ developing at 622 nm λ_em_ at neutral pH, which was further accentuated under alkaline conditions (Fig 1). Increasing the pH caused a corresponding increase in the maximum fluorescence intensity from 77.74 (units) in the acidic sample to 146.13 at neutral pH and 232.69 at alkaline pH. It was however in the 48 hour old samples that the most distinctive shift from peak A_410:634_ at acidic pH to peak B_398:622_ at alkaline pH was evident (Fig 2). As with the 24 hour sample, peak fluorescence intensity also increased with pH at 48 hours. Notably, however, at alkaline pH the maximum value of 121.34 (units) measured for peak B_398:622_ exceeded that for peak A_410:634_ at 110.37 (units). The suspensions of *P*. *intermedia* at 72 and 96 hours, displayed similar trends as pH increased, although there was a general decrease in fluorescence values with respect to time when compared to younger cultures (Figs 3 and 4). In all samples, fluorescence excitation brightness values increased as pH increased, whilst peak fluorescence decreased with increasing culture age at equivalent pH.

Notwithstanding the increase in fluorescence intensity at all wavelengths with pH, there was a notable decrease in the relative prominence of the J-band at all time-points in the EEMs (Figs 1–4). The J-band was evident as a relatively defined peak at 410 nm λ_ex_; 700 nm λ_em_ at acidic pH. With increasing pH, the fluorescence intensity of the J-band increased in proportion to that observed with peak A_410:634_, however a new broader, more intense peak emerged at 390–410 nm λ_ex_; 670–690 nm λ_em_ at neutral and alkaline pHs.

**Fig 5:**
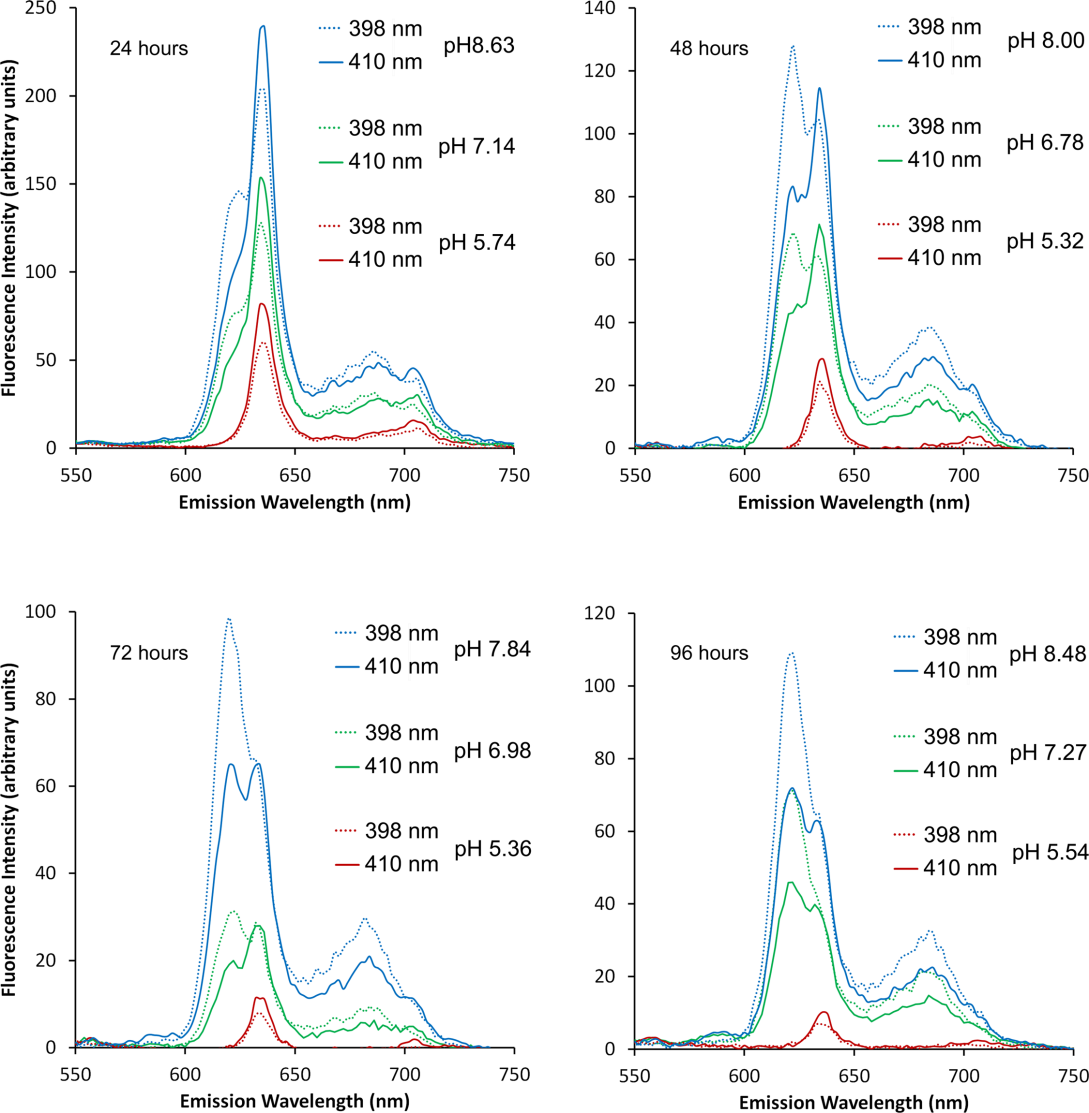
Fluorescence emission spectra at excitation wavelengths of 410 nm (Peak A) and 398 nm (Peak B) in suspensions of *P*. *intermedia*. Spectra captured from different culture ages at ‘acidic’ (red), ‘neutral’ (green) and ‘alkaline’ (blue) pHs.

When emission spectra at the specific excitation wavelengths associated with peaks A (410 nm λ_ex_) and B (398 nm λ_ex_) were isolated from the EEM, their respective emissions at 634 and 622 nm were clearly discernible under certain conditions (Fig 5). Excitation at 410 nm produced the typical emission spectra associated with PPIX at 634 nm with secondary peaks at 684 nm and 704 nm (J bands). As in the EEMs, a ‘shoulder’ could be seen forming at 622 nm λ_em_ with increasing pH 410 nm λ_ex_; however this was more prominent at 398 nm λ_ex_. The differentiation between peak A_410:634_ and peak B_398:622_ increased with increasing culture age. In 48 hour cultures and older at neutral pHs and above, peak B_398:62_ was more intense than peak A_410:634_ (with the exception of ‘neutral’ at 48 hours which were roughly equal). At acidic pH, there was no evidence of peak B_398:622_ at any time point.

**Fig 6:**
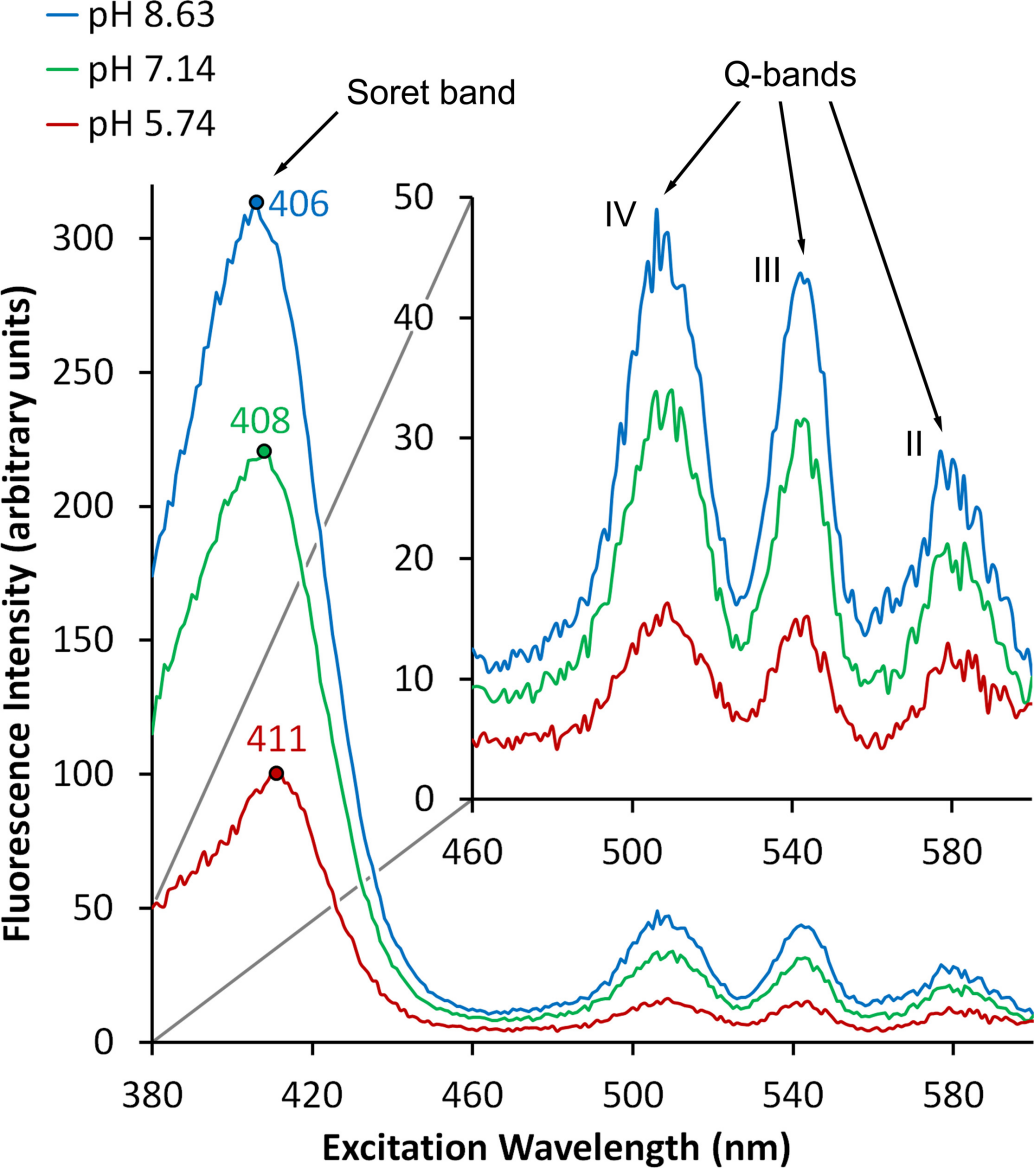
Excitation spectra at an emission wavelength of 634 nm in a 24 hour old suspension of *P*. *intermedia*. Spectra focussing on the Q-bands at different pHs. Inset: Expanded view of the Q-band region.

Scans which focussed on the emission spectra revealed Q-bands IV, III and II at 505, 542 and 580 nm respectively (Fig 6). Analysis of the peak values suggests that the changes to fluorescence emissions in the Soret band were proportionally reflected within the Q-band region, although the signal-to-noise ratios at these longer excitation wavelengths were too high to allow accurate measurements. There was also a measureable hypsochromic shift in the Soret band with increasing pH; 411 nm at pH 5.74, 408 nm at pH 7.14, 406 nm at pH 8.63.

**Fig 7:**
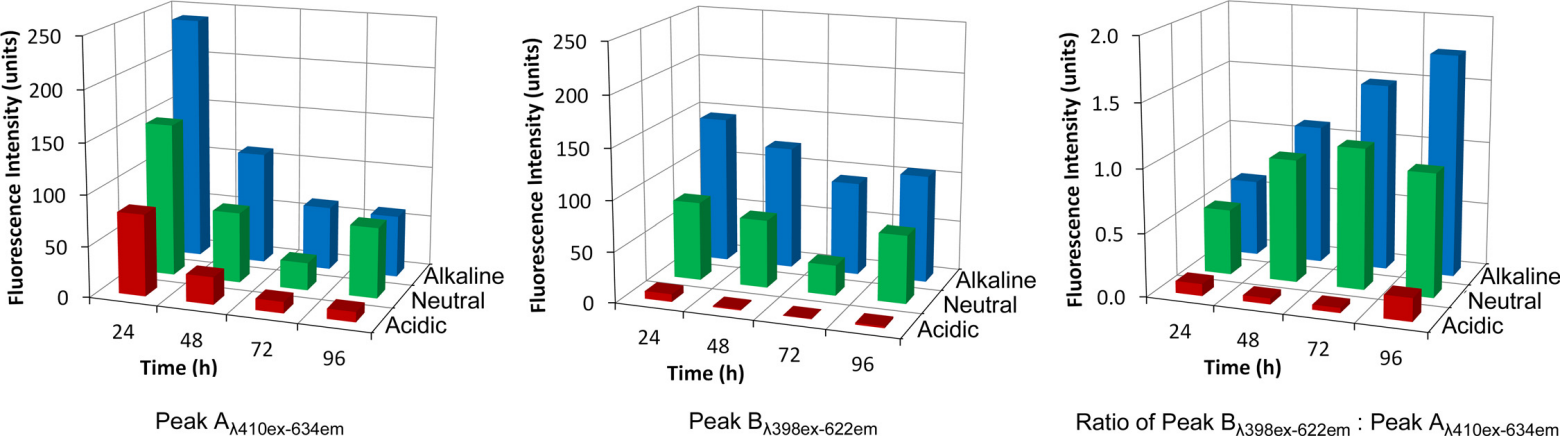
Peak fluorescence values at different pH over time in suspensions of *P*. *intermedia*. a) Peak A_410:634_. b) Peak B_398:622_. c) Ratio of Peak B_398:622_ / Peak A_410:634_.

A comparison of the maximum fluorescence values from the 2 nm wide data arrays for peak A_410:634_ and peak B_398:622_ revealed trends in both culture age and pH (Fig 7). For peak A_410:634_, fluorescence increased as pH increased whilst fluorescence decreased as culture age increased. With regard to peak B_398:622_ fluorescence, the time related trends were much less pronounced than those relating to pH. However, the distinctions between the acidic and the other samples were very clear. Ratiometric analysis between peak A_410:634_ and peak B_398:622_ indicated that fluorescence spectrophotometry can distinguish between acidic and neutral / alkaline pH, but not between neutral and alkaline conditions in young cultures (24 and 48 hours). In older cultures (72 and 96 hours) it was possible to distinguish between all of the pH states by ratiometric analysis.

The fluorescence measurements presented as excitation emission matrices are available in S1 File.

## Discussion

A bacterium within the genus *Prevotella*, *P*. *intermedia*, was selected as a model oral microorganism for this study on the basis of the ubiquity of this genus within the oral cavity [27], its strong fluorescence emissions [28] and copious production of protoporphyrin IX [2]. These experiments were not intended to accurately model the fluorescence of dental plaque, but rather to offer insight into what effect oral pH might have upon the photophysical properties of bacterial fluorescence under QLF illumination conditions. Studying a single-species culture obviated the potential confounding effects of the reported interspecies relationships, such as that reported between *Porphyromonas gingivalis* and *Peptostreptococcus micros*, which may also contribute to plaque fluorescence [29]. The emission spectrum at 410 nm λ_em_ was indicative of that observed when protoporphyrin IX is in the monomeric form. Whereas the parameters of peak B_398:622_ are associated with dimers and higher aggregates of protoporphyrin IX [19,30], although this assumption is confounded by the presence of water-soluble porphyrins, specifically coproporphyrin III in the case of *P*. *intermedia*, which has similar fluorescent properties [3,23]. The interference between the fluorescence emissions of protoporphyrin IX at 632 nm and coproporphyrin III at 622 nm has already been reported [31]. Likewise, a study of the fluorescence of dental calculus was also able to resolve two excitation peaks, at 398 and 405 nm, and attributed these to the presence of ‘at least two porphyrin derivatives’ [32]. Furthermore, 622 nm fluorescence emission has also been reported in carious lesions at 405 nm excitation [33]. The observed shifts in the fluorescence intensities from peak A_410:634_ towards peak B_398:622_ with culture age could conceivably be indicative of a shift in the amount and relative proportions of protoporphyrin IX and coproporphyrin III over time [34]. However, there is no credible mechanism by which pH adjustment could affect the proportions of protoporphyrin IX and coproporphyrin III in otherwise identical aliquots.

A previous study [10] used an aqueous solution of protoporphyrin IX to study the effects of pH on aggregate formation. In their ‘intermediate pH range’ (pH 3–7; broadly equivalent to ‘acidic’ and ‘neutral’ in the present study) they reported the formation of prolate vesicles which formed ‘face-to-face’ dimers above pH 8 (equivalent to ‘alkali’ in the present study). Their results corroborate those of the present study in that the peak absorbance and emission values at high pH were greater than at ‘intermediate’ pH. An earlier study [35] similarly reported the formation of micelles at high pH with fluorescence emission spectra. They also reported a hyspochromic shift at λ_ex_ 397 nm from pH 3.4 (λ_em_ 634 nm) to pH 11.3 (λ_em_ 620 nm). Their titration experiments of pH against fluorescence showed the transition between 620 and 634 nm emissions occurred at ~pH 7.5. More recently, a particularly pertinent paper discussed two fluorescence emissions from PPIX in gliomas (cancer of glial tissue; brain or spine) at 620 and 634 nm. These two emission peaks were attributed to the effects of pH upon PPIX [36].

The excitation spectra obtained using a wide excitation slit (Fig 6) revealed a hypsochromic shift of 2–3 nm with increasing pH from acid to neutral to alkaline which is commensurate with the 2 nm shift reported in 5,10,15,20-tetrakis(4-N-methylpyridyl) porphyrin tetratosylate salt between its mono-deprotonated and free base forms [37]. Porphyrins are amphoteric due to the porphine core being a large hydrophobic moiety with hydrophilic propionate side chains. The acid-base equilibria of porphyrins range from double protonation, mono-protonation, free base, mono-deprotonation to double deprotonation. The pH range employed in the present study may initiate a similar change in the protonation state of protoporphyrin IX which can affect its absorption peak, for example, deprotonation can result in a hypsochromic shift [17]. Results suggest that the pH range found in the oral cavity could be sufficient to illicit measurable changes in bacterial fluorescence which could interfere with the interpretation of QLF measurements.

By harvesting colonial material from the same agar plate over the course of time it was hoped that variations between samples would be minimised and that any differences would be down to culture age rather than differences in the growth medium or the cell density /growth phase of the inoculum. Single pH measurements were taken for each sample since the pH at the point of EEM capture was considered more important than quantifying the change in pH *per se*. It is unclear how the timescales of culture ages utilised in the present study (up to 96 hours) relate to the maturation of a population of *Prevotella* spp., or indeed any bacterium, growing within a multispecies, pseudo-steady-state, oral biofilm. Nevertheless, it can be assumed that protoporphyrin IX accumulation increases as a function of time in undisturbed dental plaque [2]. In general, the amount of fluorescence emitted at peak A_410:634_ or peak B_398:622_ decreased with culture age, possibly as a result of shielding of the excitation light by the strongly absorbing porphyrins as their concentration increased [38].

The pH range employed in the present study (5.32–8.63) falls within the pH range observed in dental plaque; from that found in cariogenic plaque following a glucose rinse (pH 4.66) [39] to that found within a periodontal pocket (pH 8.68) [40]. This pH range is not problematic for this strain of *P*. *intermedia* since it is capable of growth in acidic conditions as low as pH 5 [41] and has a terminal pH of 5.42 (in liquid culture containing glucose), although it is killed at pH 4.5 [42]. The terminal pH of *P*. *intermedia* growing on blood agar has been reported as being 6.08 [2], although the ‘acidic’ samples obtained in the present study suggest that this may be as low as 5.32 (Table 1).

Changes in fluorescence emissions over time have been previously reported in bacteria commonly found in the human gastrointestinal tract [15]. This study revealed a shift in the fluorescence emissions of *Haemophilus parainfluenzae*, following excitation at 370–440 nm, from a peak at 635 nm to 618 nm over a period of seven days. The mechanism underlying this transition was attributed to the formation of different porphyrins over time; initially protoporphyrin IX and latterly water-soluble porphyrins. This study also reported another fluorescence phenomenon which could manifest as a shift in fluorescence spectra; the formation of water-soluble porphyrins which occurs as a result of protoporphyrin IX degrading to photoprotoporphyrin following irradiation due to photobleaching [43]. Finally, there is also a metabolic route for the conversion of coproporphyrinogen III to protoporphyrinogen IX and then protoporphyrin IX under anaerobic conditions via the enzymes coproporphyrinogen dehydrogenase (EC 1.3.99.22; *hemN*) and protoporphyrinogen IX dehydrogenase (EC 1.3.5.3; *hemG*) [44].

## Conclusions

These findings support the position that the red fluorescence of dental plaque could be affected by its maturity [45] and that pH has a modulating effect. The spectral shifts and increase in fluorescence with increasing pH will be superimposed upon any other changes in fluorescence that may be due to community composition / plaque maturity and plaque metabolism. The shift in fluorescence emission from 635 nm to 622 nm with culture age in the current experiment corresponds with what has been previously reported as a change from ‘red-orange (moderate)’ to ‘orange (weak)’ [4]. Future work in this field should consider using multispecies models of oral biofilm to better understand the relevance of the reported pH-related phenomena and the ramifications that this may have for diagnostic tools which measure plaque fluorescence, such as QLF.

## Supporting Information

S1 File Contains all of the fluorescence values which comprise the EEM for each time point (i.e. 24h, 48h, 72h and 96h) and at each pH (i.e. acid, neutral and alkaline). (ZIP)
